# Supplementary figures and images for: Rapid, without focus stacking, 3D photogrammetric digitization of cockroaches
Source: PLoS One. 2025 Dec 1;20(12):e0336893. doi: 10.1371/journal.pone.0336893 (PMC12668519; doi:10.1371/journal.pone.0336893)

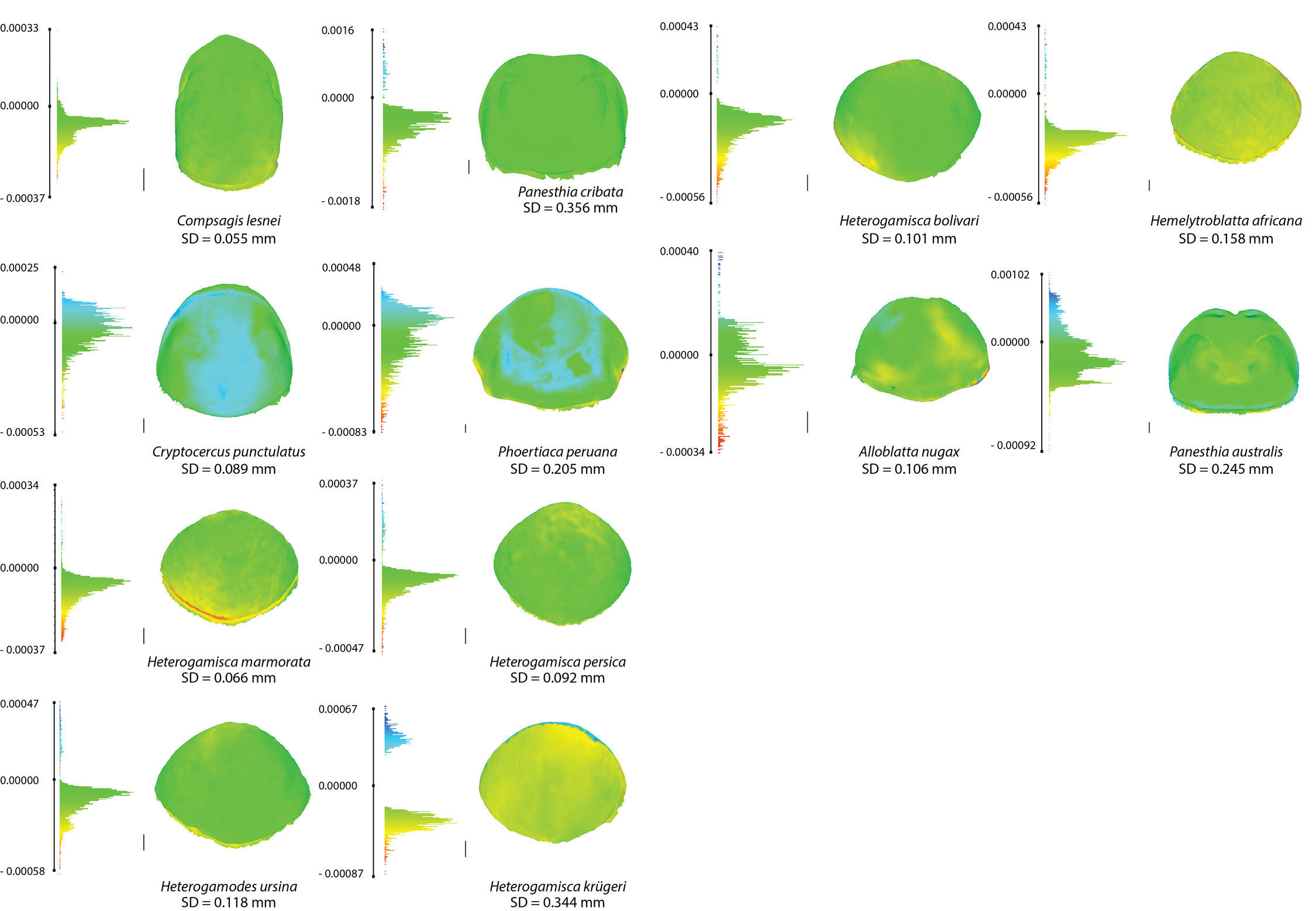

Supplement: S1 Fig — SD = standard deviation; scale bars = 1 mm. (TIF) [file pone.0336893.s001.tif]
