# Supplementary material for: Rapid, without focus stacking, 3D photogrammetric digitization of cockroaches
Source: PLoS One. 2025 Dec 1;20(12):e0336893. doi: 10.1371/journal.pone.0336893 (PMC12668519; doi:10.1371/journal.pone.0336893)
Supplement: S1 Table — Species are ordered according to the absolute value of average distance between meshes. (DOCX) [file pone.0336893.s002.docx]

| Species | Minimum distance | | Maximum distance | Median distance | Average distance | Standard deviation |
| --- | --- | --- | --- | --- | --- | --- |
| *Cryptocercus parvus* | | -0.280 | 0.179 | 0.007 | -0.006 | 0.087 |
| *Alloblatta nugax* | | -0.345 | 0.400 | -0.021 | -0.012 | 0.106 |
| *Cryptocercus punctulatus* | | -0.533 | 0.250 | -0.038 | -0.046 | 0.089 |
| *Compsagis lesnei* | | -0.373 | 0.326 | -0.073 | -0.084 | 0.055 |
| *Heterogamisca marmorata* | | -0.365 | 0.341 | -0.083 | -0.095 | 0.066 |
| *Phortioeca peruana* | | -0.826 | 0.484 | -0.058 | -0.096 | 0.205 |
| *Heterogamisca persica* | | -0.475 | 0.371 | -0.118 | -0.120 | 0.092 |
| *Heterogamodes ursina* | | -0.592 | 0.467 | -0.122 | -0.133 | 0.118 |
| *Panesthia australis* | | -0.923 | 1.023 | 0.101 | 0.147 | 0.245 |
| *Heterogamisca bolivari* | | -0.558 | 0.430 | -0.134 | -0.154 | 0.101 |
| *Panesthia cribrata* | | -1.790 | 1.568 | -0.197 | -0.178 | 0.356 |
| *Heterogamisca krügeri* | | -0.831 | 0.666 | -0.344 | -0.183 | 0.344 |
| *Hemelytroblatta africana* | | -0.878 | 0.728 | -0.345 | -0.352 | 0.158 |
