# Supplementary material for: Rapid, without focus stacking, 3D photogrammetric digitization of cockroaches
Source: PLoS One. 2025 Dec 1;20(12):e0336893. doi: 10.1371/journal.pone.0336893 (PMC12668519; doi:10.1371/journal.pone.0336893)
Supplement: Attachment — Submitted filename: Comments to the authors.pdf [file pone.0336893.s004.pdf]

Berger and colleagues meticulously developed an optimal protocol for reproducing small objects like cockroaches. I personally enjoyed reading the paper and truly appreciated the attention to detail, the table on incurred costs, and all the effort and dedication involved in refining this approach without utilising photo stacking.

I have a question stemming from curiosity: although the primary focus was on the first dorsal thoracic segment (pronotum) and all attention was directed towards the dorsal features of the insects, I wondered whether additional elements, particularly on the ventral side, could also be included in the 3D model. Was this a limitation of the method, or do you believe that adjustments, such as positioning the insect higher on the pedestal (without that brown section of the equipment obscuring the ventral side, as illustrated in your Figure 2 Step 3), could be made to capture the ventral features as well?
