# Supplementary material for: Rapid, without focus stacking, 3D photogrammetric digitization of cockroaches
Source: PLoS One. 2025 Dec 1;20(12):e0336893. doi: 10.1371/journal.pone.0336893 (PMC12668519; doi:10.1371/journal.pone.0336893)
Supplement: Attachment — Submitted filename: Response to Reviewers.docx [file pone.0336893.s007.docx]

**Revision letter for manuscript ID PONE-D-24-54510 entitled “Rapid, without focus stacking, 3D photogrammetric digitization of cockroaches”**

Dear *PLoS One* editorial board members, and reviewers,

We thank you for giving us the opportunity to revise and resubmit our study. We also thank the five reviewers, who provided constructive comments to improve our manuscript. We have carefully considered all comments and suggestions. Among the most important changes, we have now revised the manuscript by addressing the following main points raised by the reviewers:

1) We have improved the contextualization of this work and expanded on how it compares with other works (reviewers #2 and #5).
2) We have clarified several aspects of the method used (reviewers #1, #2 and #5).

In the appended copy of the reply letter, we provide a point-by-point response to the reviewers' comments, with our responses in green font. We hope this revised manuscript will make a worthy contribution to *PLoS One*.

Yours sincerely,

Juliette Berger, on behalf of the co-authors

**Reviewer #1:**

(1) It seems AF-S DX Micro NIKKOR 40 mm f/2.8G lens is auto focus lens with maximum focal length 40mm. In this case, focal length might have changed if authors used auto focus mode. Did authors fixed the focal length or used auto focus mode?

If focal length was fixed somehow, did authors conduct camera calibration? What are the interior orientation parameters (principal point offset, focal length, coefficient of radial lens distortion, coefficient or decentering lens distortion)? The camera calibration information should be described with associated condition equation.

There was no calibration step as the camera and its focal were automatically recognized by Metashape as indicated in chapter 4 of Metashape’s manual (i.e. *Reference and calibration*). We have now clarified this point.

If authors used auto focus mode for each scene, how did authors address these varying focal lengths problem? How did authors conduct camera calibration with difference focal lengths for each scene? Also, please describe why the authors didn't use fixed focal length lens in this case.

We used a fixed focal length with a manual focus. It is now clarified in the ***Photogrammetry set-up installation*** section.

(2) The description about exterior orientation (EO) parameters results (position and attitude (rotation) of camera) are not sufficient. Please describe EO parameters results more in details.

Right, thank you. We have now expanded this section as follows: “The internal and external orientation (IO & EO) parameters of the images were calculated automatically in Metashape. Indeed, during the photo alignment procedure, Metashape estimates the interior and exterior camera orientation parameters, including nonlinear radial distortions. To successfully estimate the camera orientation parameters, the information on approximate focal length (pix) is required. This information is extracted automatically from the EXIF metadata.”

(3) The precision (post adjustment statistics) and accuracy of triangulation results were not included in the manuscript. Please include them.

Right. They are now provided in a Supplementary spreadsheet document.

Thank you very much for your comments, which notably helped to clarify our workflow.

**Reviewer #2**:

Overall comment:

This study provides valuable data on the use of photogrammetry as a tool for obtaining morphometrics from specific structures of insects, in this case cockroaches. The study's strength lies in providing an accessible, streamlined and feasible workflow that should improve research efforts in the field of entomology while reducing costs and optimizing processing time. However, some aspects of the Methods section require additional clarification for readers of PLoS One to fully appreciate the robustness of the approach. Additionally, several minor instances throughout the manuscript could use clarifications and rephrasings. Overall, I believe these points can be addressed by a thorough revision of the manuscript. Detailed comments and suggestions are provided below.

We sincerely thank the reviewer for their positive and encouraging feedback.

Specific comments:

ABSTRACT

L16-21: This contextualization is important and I find it lacking in the beginning of the Introduction. Consider briefly expanding your first paragraph to fully contextualize readers about the importance of such collections.

We have expanded the first paragraph accordingly.

L21: " ...insect specimens, remain to be improved” – change to “remains”.

Done.

INTRODUCTION

L45: It does not limit, but warrants manipulation prevention. Please, consider rephrasing.

We have rephrased accordingly.

L53: I’m not sure what you mean by “keep them alive”. You mean the preservation of the NHC? Please, clarify and rephrase.

We have removed this part while rephrasing this sentence.

L56: The paper would gain from stating which other purposes Photogrammetry is also valuable for. For instance, you can cite many other advantages, like being able to maintain a digital register than can be revisited, being able to access precise metrics from different view points, among others.

Right, thank you. It has been done.

L62: I do not understand “which can the thorny for type specimens”. Please, clarify.

We have now clarified this sentence.

L85: Consider improving the flow of the sentence when citing the cost of rapidity.

Done.

L89: Is this akin to a Macro photography? Consider better explaining how the focus on the region of interest eliminates the need for focus stacking. This is particularly important because that’s one of the main aspects of you study, and I feel more time should be invested in explaining how exactly this is achieved.

Yes, this comparison may be valid. We have clarified this part.

L92-93: Consider rephrasing to “...this pipeline can produce high-quality 3D models of the structure of interest that realistically capture...”

Done, thank you.

MATERIALS AND METHODS

L100: This sentence is na Objective and should be stated in the last paragraph of the Introduction, expanded in more details.

It is actually a longer-term research objective, not the final goal of this methodological paper, so we think that it does not need to be expanded in the introduction of this manuscript. We have now clarified this.

L101-103: Consider replacing the word “tool” for “structure” here and elsewhere. Also, the sentence would benefit by stating the focus on the body part in the beginning instead of the end.

Ok, done.

L110: “Cockroaches”, in the plural.

Right, thank you.

L119: Usually we state numbers like these in the numeric form, i.e. 26 and 15. Small numbers, <10, are correctly stated throughout.

Right. Because the first number (sixteen) was the first word of the sentence (and thus not written in numeric form), we wrote the following numbers likewise for the sake of consistency. We have nonetheless changed as suggested.

L120: I had trouble understanding what you were referring as challenging here. Only when I advanced in the text I understood. Please, consider clarifying this to make it easier for readers to understand exactly that the presence of bristles, reflection and black coloration are the challenges cited.

Ok, it is now clarified.

L125: The manuscript would benefit by keeping units consistent. Consider converting to mm.

You are right, thank you.

L129: Here it is clearer which are the conditions that are challenging for photogrammetry, but you are talking about X-Rays? It is confusing; please, clarify.

This is correct. We have clarified this.

L138: Replace “shots” for “images” of “pictures”.

Done.

L146-147: Only citing the portability of the Godox LST40 is irrelevant here. If portability is an important factor, as stated in the Results and Discussion, please clarify that the whole set-up is portable.

All right.

L168 Table 2: The price stated for Metashape here is different than the one in Results and Discussion (830 USD). Please, clarify.

Right, thank you. It is now clarified.

L173: Brightness of the specimen? You mean brightness of the lighting conditions for each specimen? Please, clarify.

We meant the reflection of light on the specimen. We have clarified the sentence.

L208-221: This is an important step in generating high-quality models and I feel should be improved. It seems to be missing the step on integrating the masks generated in Photoshop to the workflow in Metashape. It would be important for readers to be able to fully reproduce the settings of this pipeline with full details.

Yes, we have now clarified this part.

L267: You mean 96 GB of RAM?

Right, thank you.

RESULTS AND DISCUSSION

L318: As I mentioned, there is a discrepancy in the values informed.

L319: This sentence in weirdly placed here. Consider rephrasing (e.g. the photographic set-up is portable, inexpensive and provides a fast workflow).

L324: Consider replacing “issues” for “limited”.

L327: Specify that you refer to “focus” stacking for consistency.

L367: Avoid superlatives such as 'ultra-high' when not referencing specific parameters from photogrammetry-based software. Consider stating that 3D models are of high quality, in a more conservative manner.

L376: Change to “PCA”.

L379: “Correspond” to “corresponded”.

L388: Consider rephrasing to “are of good enough quality”.

L318-L388: Thank you for your thorough proofreading and suggestions. We have rephrased accordingly.

**Reviewer #3**:

Berger and colleagues meticulously developed an optimal protocol for reproducing small objects like cockroaches. I personally enjoyed reading the paper and truly appreciated the attention to detail, the table on incurred costs, and all the effort and dedication involved in refining this approach without utilising photo stacking.

Thank you for this highly positive feedback.

I have a question stemming from curiosity: although the primary focus was on the first dorsal thoracic segment (pronotum) and all attention was directed towards the dorsal features of the insects, I wondered whether additional elements, particularly on the ventral side, could also be included in the 3D model. Was this a limitation of the method, or do you believe that adjustments, such as positioning the insect higher on the pedestal (without that brown section of the equipment obscuring the ventral side, as illustrated in your Figure 2 Step 3), could be made to capture the ventral features as well?

It is true that the sample holder (i.e. the ‘brown section of the equipment’) obscures the ventral side. But the ventral side was nonetheless reconstructed here, even if not used (like the other parts of the specimens, which were not analyzed either). As suggested, a different positioning of the specimens would indeed improve the quality of the antero-ventral part of the models. Even with such an adjustment, however, the 3D reconstructions might still be suboptimal, mainly because if the legs are extended, they increase the depth of field. This is a limitation of this rapid method, as discussed in the ***Limitations*** section.

We appreciate your interest in our manuscript and the time you took to review it.

**Reviewer #4**:

The manuscript "Rapid, without focus stacking, 3D photogrammetric digitization of cockroaches" presents an innovative methodology for digitizing insect specimens, specifically cockroaches, for morphometric analysis. The authors propose a photogrammetric pipeline that excludes the time-consuming process of focus stacking, which is traditionally used to increase the depth of field in photogrammetry, particularly for small objects. The authors present a well-structured study and apply a robust methodology to address the gap in the adoption of photogrammetry applications. Only minor details need to be addressed.

Thank you for reviewing our work and highlighting its novelty.

Including error values in the scale bars will further support the absence of significant biases between the two techniques used in your work.

Thank you for this suggestion. This information is now provided in our new Supplementary spreadsheet document.

The hyphen ("-") appears to be a typo or error (e.g., in lines 24 and 28). Correct this inconsistency throughout the document as needed.

It is neither a typo or an error but an Em Dash, which can function like a comma or parenthesis. See for instance https://www.merriam-webster.com/grammar/em-dash-en-dash-how-to-use

Ensure consistency in word choice by selecting either "modeling" or "modelling" and applying it uniformly.

Right, it is now done.

**Reviewer #5**:

This study shows the advantages of a photogrammetric workflow to generate high quality 3D models of objects as small as insects (cockroaches). In general, I consider the work publishable after review and correction of some points:

The introduction fulfills its purpose by showing the reader the benefits of this workflow tested on cockroaches. However, I think that the introduction can be improved by including the following bibliography.

- Beth et al., (2012). Whole-drawer imaging for digital management and curation of a large entomological collection.

- Muthu et al. (2023). Towards end-to-end automatic insect handling and insect scanning.

- Thanh-Nghi Doan (2024). A low-cost digital 3D insect scanner.

- Mathys et al. (2024). Sphaeroptica: A tool for pseudo-3D visualization and 3D measurements on arthropods.

Thank you very much for these suggestions. Your comment echoes that of R#2, who asked us to provide a little more context for our study. We have now strengthened the introduction, citing the studies you suggested, as well as others we found to be relevant.

Line 125: Standardize the unit of measurement of the specimens, either in cm or mm. In line 125 they are in cm and in line 117 in mm.

Done.

Line 183: Mention what angles were used in previous or similar studies.

The purpose of taking pictures from different angles is to maximize overlap between images to ease the alignment step and produce high-quality models. It depends on several parameters like the size of and distance to the specimens, and of the region of interest (ROI). Thus, it does not make much sense to compare these angles across studies. In our case, because our ROI was the pronotum (in the anterior part of the specimens), the angles chosen were 90°, 130° and 160°. They would have been quite different if the ROI was the tip of the abdomen for instance. We have now further clarified this point.

Line 231: Almost all photogrammetric processing with Agisoft Metashape software was done with high values, except for photo alignment. Why did you choose a medium value instead of a high value? There are other steps that are more time consuming than photo alignment (e.g. building a dense cloud). Also, increasing the alignment to "high" may improve the accuracy of your measurements.

We followed Ströbel et al. (2022), whom we cite at the beginning of the workflow, in making this methodological choice that speeds up the process without compromising the quality of the models. We also provide an additional spreadsheet demonstrating the high accuracy of our measurements. Any further increase in accuracy would be negligible for our analyses, while extending the length of the process, which goes against one of the main motivations behind our work.

Line 254: How many markers were placed on the photos? Were the measurements obtained from this scale assignment to the 3D models subsequently validated by measuring the specimens?

We used two markers. And yes, the scale was validated by measuring the specimens. We have clarified this, thank you.

Line 267: Correct 96 Go by 96 GB

Ok, done.

Line 311: Compare the results with the same or similar studies using photogrammetry.

Yes, we agree, and that is indeed what we have done. To the best of our knowledge, no photogrammetry protocol adapted for small objects does not use focus-stacking. We therefore compare our workflow with those of others, all of which use focus-stacking, such as Doan & Nguyen 2024, whose study is the most similar to ours. These comparisons are reported in the sections “**3D entomology models without focus stacking for comparative morphology**” and “**Limitations: a heterogeneous model quality**”, which emphasize respectively the strengths and limitations of our approach in comparison to others.
